# Inactivation of *Listeria monocytogenes* and *Salmonella* spp. in Milano-Type Salami Made with Alternative Formulations to the Use of Synthetic Nitrates/Nitrites

**DOI:** 10.3390/microorganisms10030562

**Published:** 2022-03-04

**Authors:** Elena Dalzini, Daniela Merigo, Alessia Caproli, Paola Monastero, Elena Cosciani-Cunico, Marina-Nadia Losio, Paolo Daminelli

**Affiliations:** 1 National Reference Centre for Emerging Risks in Food Safety, Istituto Zooprofilattico Sperimentale della Lombardia e dell’Emilia Romagna “B. Ubertini”, 20133 Milan, Italy; daniela.merigo@izsler.it (D.M.); alessia.caproli@izsler.it (A.C.); paola.monastero@izsler.it (P.M.); elena.coscianicunico@izsler.it (E.C.-C.); marinanadia.losio@izsler.it (M.-N.L.); paolo.daminelli@izsler.it (P.D.); 2 Food Control Division–Food Safety Department, Istituto Zooprofilattico Sperimentale della Lombardia e dell’Emilia Romagna “B. Ubertini”, 25142 Brescia, Italy

**Keywords:** challenge test, fermented meat, traditional products

## Abstract

During the manufacture of Italian salami, a traditional meat product, a sequence of hurdles like meat fermentation, air-drying, and long ripening processes are generally sufficient to inhibit the growth of most pathogens. Furthermore, Italian salami are traditionally produced by adding synthetic nitrates/nitrites to raw meat with safety and technological aims, even if controversial opinions about their use still remain, particularly in relation to the consumer demand for natural food products. In this context, the aim of the study was to investigate the inactivation of *Listeria monocytogenes* and *Salmonella* spp. during the manufacturing process of Milano-type salami made with different formulations to evaluate the contribution of the hurdles and the vegetable or synthetic additives on the inactivation of pathogens. Thus, a challenge study was performed dividing ca. 400 kg of Milano-type salami batter into three batches: Batch (A) without nitrates/nitrites; Batch (B) with vegetable nitrates, and Batch (C) with synthetic nitrates/nitrites. The batches were separately inoculated with *L. monocytogenes* and *Salmonella* spp. and the pathogens’ survival was evaluated during the fermentation, draining, and 70-day ripening of the Milano-type salami. The pathogen counts decreased in all tested conditions, even though the highest inactivation of *L. monocytogenes* and *Salmonella* spp. (*p* < 0.05) was observed when nitrates or nitrites were added to the batter. This study shows how the safety of these products cannot exclude the aspect of the hurdle technology during the process, which plays a major role in the reduction of pathogens, but additives like nitrates and nitrites allow for a greater margin of safety. Thus, further studies are needed to validate the use of natural compounds as alternatives to conventional preservatives in meat products. These results may provide new information to support food business operators in producing traditional foods with alternative preservatives and competent authorities in verifying the safety of the products made with natural compounds, and to control the process parameters responsible for the synergistic effect against pathogens such as *L. monocytogenes* and *Salmonella* spp.

## 1. Introduction

Italian salami are typical fermented dry or semi-dry sausages (FDSS) traditionally made with minced pork meat and fat, often with added spices, herbs, starter cultures, and preservatives [1]. Foodborne pathogens like *Listeria monocytogenes* and *Salmonella* spp. may occur in these products via contaminated raw meat, ingredients, processing equipment, and/or as consequence of post-processing contamination [2,3,4,5]. 

During the process, salami are formulated using hurdle technology, a concept described by Leistner [6] as a sequence of antimicrobial barriers leading to safer and more stable products [7]. The combination of meat fermentation, enabled by the addition of selected lactic acid-producing starter cultures, and air-drying and mold-ripening conditions causes a decrease in pH and water activity (a_w_) that is generally sufficient to inhibit the growth and production of the toxins of most pathogens [2,7,8,9]. Moreover, synthetic nitrates/nitrites are commonly added into the salami batter to inhibit Gram-positive spore-forming anaerobic bacteria such as *Clostridium botulinum* [10,11], and their effect also works against *Listeria* spp. and *Salmonella* spp. [12,13,14]. The use of nitrate/nitrite compounds in meat products is regulated by EU Regulation 1129 [15], with a maximum of 150 mg/kg of nitrate and 150 mg/kg of nitrite (or a maximum of 250 mg/kg of nitrate if nitrite is not added) are allowed in cured meat products. Nevertheless, their involvement in the formation of nitroso and carcinogenic compounds makes the consumption of cured meats a matter controversy [16]. The consumer demand for organic or natural food products has increased in recent years; natural foods for consumers have various meanings, including food without the addition of something considered unhealthy, or with a low content of something considered unhealthy [17]. 

In recent years, several studies were conducted on processed meat such as dry-fermented sausages to evaluate the effect of reducing nitrates/nitrites on safety and technological implications [12,18,19,20]. The main interest for the producer is to develop alternatives from natural sources (e.g., natural nitrate) and other preservation techniques that are considered to be comparatively healthier [21]. Because of this trend, the meat industry is currently focusing on developing alternative natural strategies, like the use of novel starter culture formulations associated with specific ripening conditions [8], the application of high-pressure treatments [22], and the use of vegetable extracts as sources of natural nitrates, even if the vegetable nitrates are no healthier than synthetic ones [23,24].

However, while the microbiological safety of meat products treated with synthetic nitrates/nitrites has a long history, the microbiological safety of meat processed with alternative formulations is not so well documented [25], especially in local or traditional foods. In fact, the main issue remains finding an alternative that is able to address the same antimicrobial activities directly in the foods during the process and against the pathogens. 

Thus, the aim of this study was to investigate the inactivation of *L. monocytogenes* and *Salmonella* spp. during the manufacturing process of Milano-type salami made without nitrates/nitrites or with vegetable nitrates or synthetic nitrates/nitrites. Furthermore, the quantification of the inactivation of these pathogens during the process of making salami with different recipes can provide valuable information on the process parameters and food formulations affecting the pathogens’ survival.

## 2. Materials and Methods

### 2.1. Experimental Design

**Figure 1:**
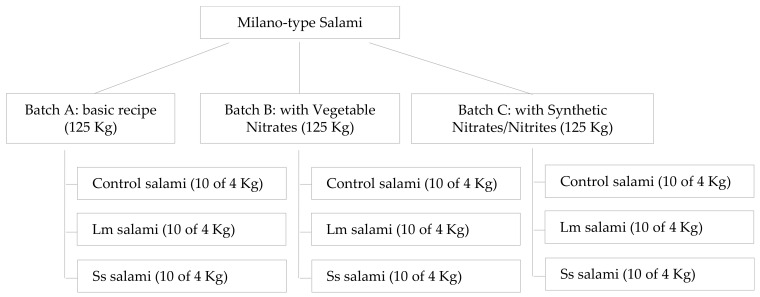
Graphical presentation of the experimental design.

Due to the large volume of batter needed (i) to obtain real-sized Milano-type salami (diameter 10 cm, length 50 cm, weight 4–5 kg), (ii) to ensure a representative volume and number of samples in which to investigate the evolution of intrinsic product properties and the pathogen behavior during the simulated process (70 days), and (iii) to compare the results obtained in each of the tested conditions (different curing additives added to the batter), the experiment was performed using three batches of batter to produce Milano-type salami (one batch for each type of curing additive). Each batch was then divided in three groups separately inoculated with *L. monocytogenes* (Lm), *Salmonella* spp. (Ss) (contaminated salami) or physiological solution (control salami) (Figure 1). Then, fermentation, drying, and ripening steps were performed according to traditional production specifications.

### 2.2. Bacteria Strains and Inoculums Preparation

Three strains of *L. monocytogenes* (ATCC^®^ 19115™ and product isolates: Lm171718 isolated from Salami and Lm171767 isolated from bacon) and four strains of *Salmonella* spp. (*Salmonella enterica* subsp. *enterica* serovar Typhimurium ATCC^®^ 6994™ and product isolates: Ss273860 isolated from fresh salami and Ss240807 isolated from pork casing and *Salmonella enterica* subsp. *enterica* serovar Derby Ss81068 isolated from sausages) were used in this study. The inocula were prepared in agreement with Dalzini et al. [26]. Briefly, each strain, kept frozen (−80 °C) in brain–heart infusion (BHI) broth (Oxoid, Milan, Italy) supplemented with 20% glycerol, was sub-cultured two times in BHI at 37 °C for 24 h. After the culture centrifugation, the pellet was washed and re-suspended in sterile physiological solution. 

Before use, the individual strains of each pathogen were combined in equal volumes in order to obtain each multi-strain cocktail and serially diluted to reach approximately 5 log CFU/g in salami batter.

### 2.3. Salami Batches

A total of 400 kg of batter was provided by a local meat company divided into three batches (125 kg of meat per batch). 

The batters were prepared and delivered to the experimental laboratory of IZSLER under refrigerated conditions (4 °C ± 2 °C) on the same day. All batches were prepared following the same recipe: lean pork shoulder (86%), pork belly (14%), dextrose (3%), salt (2.5%), pepper (0.2%), sodium ascorbate (0.07%), and microbial starter cultures (Chr. Hansen, Hørsholm, Denmark; BITEC, Ontario, Canada). To obtain different test conditions, Batch A (basic recipe) was used without supplementation in this study, while:

- Batch B contained a functional system (2.2 g/kg, made by Swiss chard juice concentrate powder and carrot juice concentrate powder) as natural source of nitrates (Frutarom Italy S.r.l., Parma, Italy);
- Batch C contained synthetic nitrates/nitrites (150/125 mg/kg).

Each batch was separately inoculated by the addition (1% *v*/*w*) of the individual multi-strain cocktail of *L. monocytogenes*, *Salmonella* spp., or physiological solution to produce contaminated or non-contaminated salami (control salami), respectively (Figure 1). 

### 2.4. Salami Process

The batter was mixed at room temperature (22 ± 2 °C) for 10 min, and then stuffed into synthetic casings to produce salami characterized by a length of 50 cm, a height of 10 cm, and an initial weight of approximately 4 kg. The resulting salami were then sprayed with *Penicillium nalgiovense* as a mold starter and the casings were perforated with fine needles to promote the drying process. The salami production process took 70 days: it included 24 h of fermentation at 24–22 °C and relative humidity (RH) of 90%; 24 h at 22–20 °C and RH 40–90%; 48 h at 20–18 °C and RH 40–90%; 48 h at 18–16 °C and RH 40–90%; and the remaining process until the end of ripening at 15–13 °C and RH 70–80%.

### 2.5. Sampling

For each batch, two technical replicates of 250 g were collected from the salami at 0 (batter), 1, 3, 4, 7, 15, 30, 45, 60, and 70 days during the process. A portion of about 5–7 cm of salami was removed before sampling and the subsequent slice (of about 250 g) was taken and minced for few seconds to homogenize the salami’s core and surface.

### 2.6. Proximal Composition and Physico-Chemical Analyses

All of the analyses were performed on the control salami. The proximal composition of the salami batter (protein, fat, salt, ashes, and moisture) was determined for each batch according to the AOAC official method 2007.04 [27] with a FoodScan™ device (FOSS Analytic, Hillerod, Denmark) that uses the near-infrared spectrophotometer system, and the nitrate/nitrite content was determined using the previously described method [28]. 

During the process, the pH was measured on 10 g of the sample using an HI 223 Calibration check^TM^ Microprocessor pH meter (Hanna Instrument, Padova, Italy) equipped with a Gel-Glass electrode (Hamilton Company, Reno, NV, USA)) and the water activity (a_w_) was measured at 25 °C with the a_w_ recorder AquaLab, series 3, Model TE (Decagon Devices, Inc., Pullman, WA, USA) in accordance with ISO 18787 [29].

The internal temperature of the control salami was constantly monitored during the process, and for each batch, the time/temperature profiles were registered using a Thermo Button 22 L data logger (Astori Tecnica s.n.c., Brescia, Italy).

### 2.7. Microbiological Analyses

Twenty-five grams of salami samples were transferred separately into plastic one-chamber filter stomacher bags (Neomed, Milano, Italy) and then homogenized 1:10 (*w*:*v*) in sterile peptone water (PW, Conda, Madrid, Spain) for 3 min using a Stomacher 400 blender (Seward Medical, London, UK). Decimal dilutions in sterile PW were then prepared. 

Mesophilic lactic acid bacteria (LAB) were enumerated in the control salami by pour plating 1 mL of appropriate dilution in de Man–Rogosa–Sharpe agar (MRSA) (Microbiol Diagnostici, Cagliari, Italy), and the plates were incubated in accordance with ISO 15214 [30].

In the contaminated salami, *L. monocytogenes* enumeration was performed according to ISO 11290-2 [31]. For the enumeration of *Salmonella* spp., appropriate dilutions were surface-plated onto Hektoen enteric agar (HEA) (Oxoid, Milano, Italy). Typical colonies were counted after the incubation of duplicate plates at 37 °C for 24 h. To verify the absence of natural contamination of raw meat, at time zero, the enumeration of pathogens was also investigated in the control salami.

The quality of each culture medium used in this study was evaluated in accordance with ISO 11133-1 [32] and ISO 11133-2 [33]. 

### 2.8. Data Analysis

The individual means and standard deviations for all analyzed parameters were determined as the average of two technical replicates at each sampling time. 

The microbial results were expressed as colony forming unit (CFU) per g and converted to log CFU per g before the means and standard deviations were calculated. Pathogen inactivation was evaluated in terms of logarithmic reductions as the difference between the counts after ripening (70 days) (N, log CFU/g) and the average of the initial inoculum level (N0, log CFU/g) (i.e., log (N/N0). With the aim of calculating the mean log reductions, we assumed the level of contamination to be equal to 5 CFU/g (log CFU/g 0.70) when the pathogen log count was below the quantification limit (10 CFU/g) after the ripening of the salami. 

The results are expressed as an average of the log (N/N0) CFU/g (means ± standard deviation) of two technical replicates after ripening.

The significant differences of the results were evaluated during the process and between the different batches by variance analysis (ANOVA) using R v 3.4.0 software (R Development Core Team) [34]. The significance was evaluated when the *p*-value was lower than 0.05 (*p* < 0.05); in this case, a Tukey’s honest significant difference (HSD) test was performed to evaluate the significant differences among the tested conditions.

## 3. Results

### 3.1. Proximal Composition and Physico-Chemical Properties

The proximal analysis tested in all of the salami batches used in this study is summarized in Table 1. The batter provided from the local meat company was characterized from a medium fat level with percentages ranging from 23.46% to 29.6%, and a salt content of between 2.04% and 2.7%. Immediately after the formulation, the batters were also tested to evaluate the presence and levels of nitrates and nitrites: Batch A did not contain quantifiable levels of these additives, showing results below the quantification limit. Concentrations of nitrates from 84 to 90 mg/kg were found in Batch B made with vegetable sources of nitrates, and nitrate concentrations from 136 to 148 mg/kg were recovered from Batch C made with synthetic nitrates/nitrites (Table 1). 

During the manufacturing process of the Milano-type salami, the temperature reached 22 °C in the salami’s core during fermentation and then decreased to 12–13 °C during the ripening time for 70 days (Supplementary Figure S1).

**Figure 2:**
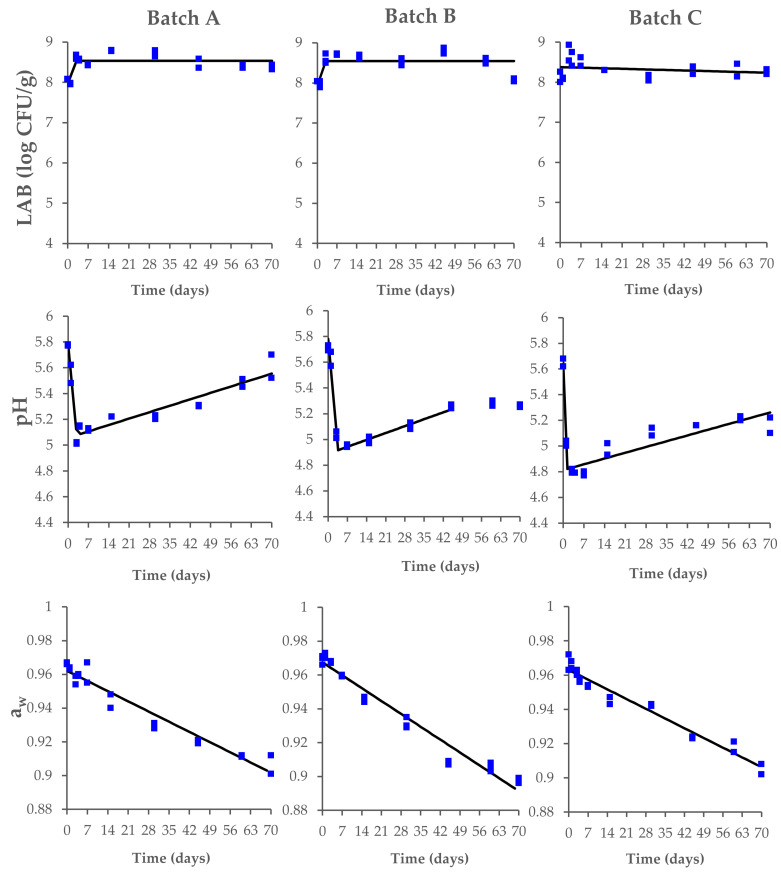
Changes in lactic acid bacteria (LAB) concentration, pH, and a_w_ values, measured during the production process of Milano-type salami made from Batch A (basic recipe), Batch B (vegetable nitrates), and Batch C (synthetic nitrates/nitrites). The observed data (square symbols of two replicate samples for each batch) were interpolated by biphasic model. The interpolation (black line) is of visual interest only.

In this context, the LAB reached the maximum concentration within the first 3 days, with values between 8.4 and 8.7 log CFU/g. The LAB fermentation caused a rapid acidification of the meat, with a decrease in pH from 5.8–5.7 to 5.0–4.8 within 3 days of the process (Figure 2). Significant changes were also observed for the a_w_ values, with a decrease from 0.97 ± 0.01 to 0.91–0.90 during the drying and ripening phases. No significant differences (*p* > 0.05) among the tested batches were found at the end of the ripening process (Supplementary Table S1). 

### 3.2. Inactivation of L. monocytogenes and Salmonella spp. during the Process

For each batch, the batter used for the preparation of the salami did not show a quantifiable natural presence of *L. monocytogenes* and *Salmonella* spp. (<1 log CFU/g).

**Figure 3:**
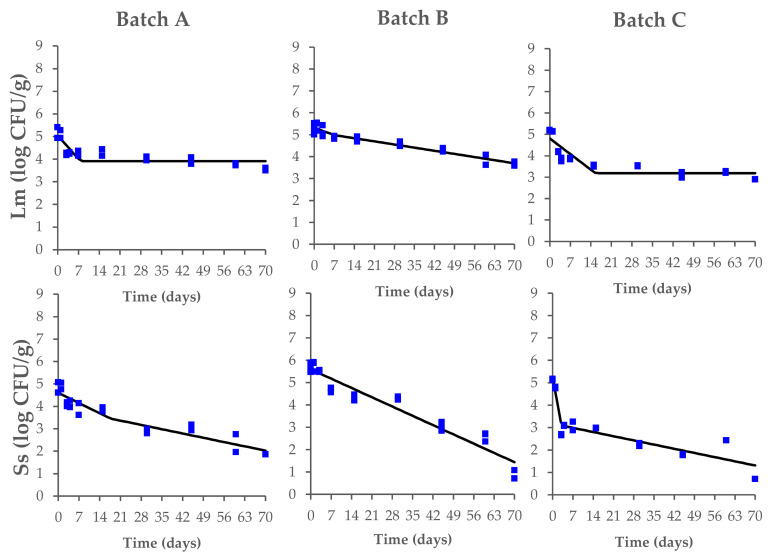
Behavior of *L. monocytogenes* (Lm) and *Salmonella* spp. (Ss) during the production process of Milano-type salami made from Batch A (basic recipe), Batch B (vegetable nitrates), and Batch C (synthetic nitrates/nitrites). The observed data (square symbols of two replicate samples for each batch) were fitted using a biphasic model. The black line is of visual interest only.

The data on the behavior of *L. monocytogenes* and *Salmonella* spp. during the fermentation, drying, and ripening of the contaminated Milano-type salami are shown in Figure 3. 

Starting from the fermentation phase, the initial counts of *L. monocytogenes* (range of 5.2–5.5 log CFU/g) decreased until they reached a concentration of 3.6 ± 0.1 log CFU/g (Batch A), 3.4 ± 0.1 log CFU/g (Batch B), and 2.9 ± 0.1 log CFU/g (Batch C) at the end of the ripening process, with significant differences between the tested batches (*p* < 0.05). In the same way, the *Salmonella* spp. counts (range of 4.8–5.6 log CFU/g) also decreased during the process until they reached a concentration of 1.9 ± 0.1 log CFU/g (Batch A) and 0.7 ± 0.1 log CFU/g (Batch B and C) (Supplementary Table S1). 

**Figure 4:**
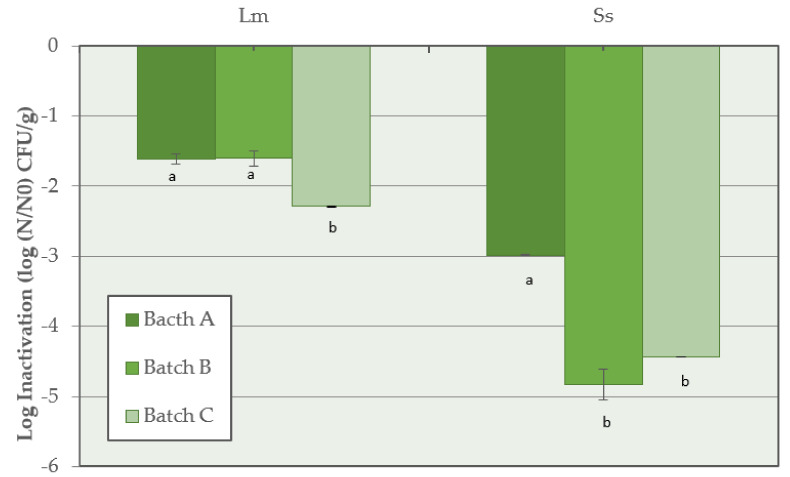
Inactivation of *L. monocytogenes* (Lm) and *Salmonella* spp. (Ss) for Batch A (basic recipe), Batch B (vegetable nitrates), and Batch C (synthetic nitrates/nitrites) of Milano-type salami ripened for 70 days. For each pathogen, means with different lowercase letters are significantly different (*p* < 0.05).

The highest inactivation of *L. monocytogenes* (*p* < 0.05) was observed when the synthetic nitrates/nitrites were added to the salami batter (Batch C) with a value of −2.3 ± 0.1 log CFU/g, compared with reductions of −1.6 ± 0.1 log CFU/g and −1.6 ± 0.1 log CFU/g calculated in Batch A and Batch B, respectively. For *Salmonella* spp., at the end of the salami development process, the highest inactivation of −4.8 ± 0.1 log CFU/g and −4.4 ± 0.1 log CFU/g was observed in Batch B and Batch C, respectively, while a lower reduction (*p* < 0.05) was calculated in Batch A (−3.0 ± 0.1 log CFU/g) (Figure 4). 

## 4. Discussion

This study investigated the inactivation of *L. monocytogenes* and *Salmonella* spp. during the manufacturing process of Milano-type salami made without nitrates/nitrites to evaluate the pathogens’ behavior using alternative ingredients or nitrate/nitrite replacements of vegetable origin.

The proximal composition tested in all of the salami batches, summarized in Table 1, shows small but significant differences (*p* < 0.05) in the protein, fat, and moisture values among the tested batches. A difference of ca. 3–7 percentage points in fat content was observed between Batch A and the other tested batches. This variability may be explained by the use of fatter pieces of meat for the preparation of the minced meat, as in Batch A, where the higher fat content seems to be directly associated with the lower moisture and protein content, as reported in previous studies [35,36]. However, the results of the protein, fat, salt, and moisture values obtained for the batches of Milano-type salami, manufactured with the different curing solutions, were similar to those reported in previous studies on Italian salami [37].

The analyses performed on the batter show the presence of nitrates/nitrites not only in Batch C, but also in Batch B, while no additives were found in Batch A.

It is known that some vegetables may contain a variable amount of nitrate, which can be converted to nitrite by the meat microflora or by additional bacteria [38]. For this reason, vegetables have the greatest potential to introduce natural sources of nitrate into natural products. Ingredients such as celery, Swiss chard, spinach, radish, and lettuce can be used by several meat processors, along with bacterial starter cultures that reduce nitrates to nitrite during the manufacturing process, to maintain the properties of typical cured meat products [21,38]. The use of vegetable extracts rich in nitrates could satisfy both the technological requirements and consumer demand for natural products, even if natural nitrates do not avoid the formation of N-nitrosamines. Moreover, the fluctuating content of nitrates in vegetable extracts could cause an overdose or an insufficient addition of nitrates. Thus, the authors suggest quantifying the amount of nitrate in the commercial formula being used before starting the salami production process.

Regarding Batch C (with synthetic nitrates/nitrites), a high percentage of nitrates was recovered—about 90–98% of the ingoing nitrate amount—in accordance with a previous study [20]. Conversely, nitrites react quickly when added to the formulation of dry-fermented sausages, showing a lower concentration of recovery compared to the added levels. In the current study, a recovery of 55–60% of the ingoing nitrite amount was observed, in agreement with Li et al. [39], who recovered between 50% and 70% of nitrites in dry-cured sausages, even though other authors reported higher values of 80% in cured meat [40] or between 71% and 82% in dry-fermented sausages [19]. 

Additives like nitrates are not unique factors controlling pathogens’ growth during the manufacturing of dry-fermented sausages; rather, a combined effect with the other hurdles is necessary to maintain control of the process.

During the first two days of the process, the pH decreased from 5.8–5.7 to 5.0–4.8 due to LAB fermentation’s acidification (Figure 2) with a temperature of 22–24 °C, and the salt concentration ranged from 2.1 to 2.7%. In accordance with the *Good manufacturing practices for fermented dry and semi-dry sausage products* proposed by the American Meat Institute Foundation [41], the fermentation process can be considered acceptable in all the tested batches. In fact, in our study, the fermentation had a degree-hours maximum of 864 (72 °F (22 °C)–60 × 72 h) that respects the criterion of <1200 degree-hours when the fermentation temperature is less than 32.2 °C (90 °F). 

During ripening, when the pH typically tended to increase, significant differences (*p* < 0.05) in the pH values were observed among the tested batches, and Batch B and C showed a pH significantly lower than Batch A (Supplementary Table S1).

The correlation between the lower pH and the presence of nitrates/nitrites is a phenomenon not always detected in similar studies. For example, Cardinali et al. [18] observed a significant effect of nitrate/nitrite concentration on pH values in Fabriano-like fermented sausages ripened for 42 days, where the pH was 5.92 ± 0.05 when the sausages were produced without nitrates/nitrites or 5.79 ± 0.08 when 150/125 mg/kg of nitrates/nitrites were added. Conversely, other authors, such as Christieans et al. [12] and Hospital et al. [19,20], reported that no significant difference of the pH was observed among dry fermented sausages made with different nitrate/nitrite concentrations, showing that the evolution of the pH does not depend on nitrate/nitrite concentration in these types of sausages. 

The changes of pH and a_w_ values obtained in our study were similar to those reported in previous studies on 27 commercial Italian salami (a_w_ mean of 0.898) [37] and in Mediterranean dry fermented sausages [12,19].

During the process of Milano-type salami production, both *L. monocytogenes* and *Salmonella* spp. counts decreased in all tested conditions (Figure 3), with a highest inactivation of *Salmonella* spp. These results are in accordance with those exhibited in other types of Italian salami [26,42,43,44,45,46]. In fact, as previously described, Gram-positive bacteria, such as *Listeria* spp., seem to be more resistant than Gram-negative bacteria to acid environments due to their defined acid tolerance resistance mechanisms [47,48,49], and this trend also emerged in the current study.

In our study, no growth of *L. monocytogenes* or *Salmonella* spp. was observed during the process, not even when the salami were produced without nitrates/nitrites (Batch A). These results, in agreement with data previously reported from other authors [9,20], show that the growth of these pathogens has not been proven during the processing of dry fermented sausages due to a combination of inhibitory factors noted as hurdles technology. According to Gounadaki et al. [50], pH and a_w_ are crucial factors for the reduction of pathogens during the process of dry fermented sausages, depending on the fermentation temperature; however, at fermentation temperatures ≥ 20 °C, as in the case of Milano-type salami, the crucial factor for *L. monocytogenes* and *Salmonella* spp. inhibition seems to be the pH decrease. Preventive measures to control the growth of *S. enterica*, *Staphylococcus aureus*, and *L. monocytogenes* during the manufacture of fermented sausages indicate the critical control points (CCPs) of the process, as proposed by Lücke [9]. These measures include: pH during butchering below or equal to 5.8; a_w_ during comminution and mixing 0.955–0.965; sugar concentration during comminution and mixing 0.3–0.5%; starter addition during comminution and mixing; fermentation at 18–22 °C; a target pH ≤ 5.3 after ca. 3 days of fermentation; and ripening at 10–15 °C with a target a_w_ ≤ 0.90. The inactivation of *L. monocytogenes* and *Salmonella* spp. observed in the current study during the manufacturing process of Milano-type salami made with these preventive measures taken into consideration show the importance of the application of hurdles technology, regardless of the use of natural or synthetic preservatives. In our study, the pH rapidly decreased, reaching 5.0–4.8 values due to LAB fermentation with a temperature of 22–24 °C, and the salt concentration ranged from 2.1 to 2.7%. However, our study shows that the highest inactivation of *L. monocytogenes* (*p* < 0.05) was observed when synthetic nitrates/nitrites (150/125 mg/kg) were added to the salami batter (Batch C). A similar trend was observed for *Salmonella* spp. inactivation, with the highest inactivation observed in the presence of vegetable nitrates or with a combination of nitrates/nitrites (Figure 4). In accordance with this finding, Christieans et al. [12] and Hospital et al. [13,14] also reported that the addition of nitrates/nitrites has an inhibitory effect on *L. monocytogenes* and *Salmonella* spp. in dry fermented sausages. 

Pathogens such as *Salmonella* spp. are known to be linked to meat-associated outbreaks, but other organisms such as *L. monocytogenes* may also be involved, even if less frequently reported [51]. Thus, it is essential to validate how the meat industry works to meet consumer demands while maintaining food safety. In recent years, controversial opinions about the use of synthetic nitrates/nitrites traditionally used to produce cured meat products still remain. Their important technological role on color, flavor, and antioxidant activities [52,53], as well as their antimicrobial effect against *Salmonella* spp., *L. monocytogenes* [12,14], *C. botulinum*, and *Staphylococcus aureus* are also noted [54]. In this context, vegetable nitrates might be proposed as an alternative to chemical and synthetic antimicrobials and antioxidants for traditional foods, but only when the strict control of the manufacturing process and the respect of the preventive measures are guaranteed.

## 5. Conclusions

The present study underlines the importance of the use of nitrates/nitrites to obtain a greater margin of safety in fermented/ripened meat products such as Italian salami. However, the safety of these products cannot exclude the respect of the preventive measures during the process, which play a major role in inhibiting the growth and survival of foodborne pathogens. Few data are available in the literature about the fate of these pathogens in traditional foods made with alternative additives to the synthetic nitrates/nitrites. Thus, further studies are needed to validate the use of natural compounds or plant extracts as alternatives to conventional preservatives in meat products. This study may provide useful information to support both food business operators and regional or national veterinary authorities to verify the safety of the products made with natural compounds, and to control the process parameters responsible for the synergistic effect against pathogens such as *L. monocytogenes* and *Salmonella* spp. 

## Figures and Tables

**Table 1:** Proximal composition and nitrate/nitrite content analyzed in the batter used to produce the Milano-type salami. Each parameter is expressed as g/100 g (proximal composition) or mg/kg (nitrates/nitrites). Values are the means ± standard deviation of two replicates samples for Batch A (basic recipe), Batch B (vegetable nitrates), and Batch C (synthetic nitrates/nitrites). For each parameter, means with different uppercase letters within a column are significantly different (*p* < 0.05) among batches.

Batch	Parameters						
	Protein	Fat	Salt (Sodium Chloride)	Ashes	Moisture	Nitrates (Sodium Nitrate)	Nitrites (Sodium Nitrite)
Batch A	16.8 ± 0.4 A	28.9 ± 1.1 A	2.1 ± 0.1 A	3.2 ± 0.1 A	51.5 ± 0.5 A	<10 *	<5 *
Batch B	17.6 ± 0.1 B	23.8 ± 0.5 B	2.6 ± 0.2 A	3.5 ± 0.1 A	55.5 ± 0.3 B	87 ± 4.2 B	5.5 ± 0.7 A
Batch C	17.8 ± 0.3 B	25.9 ± 0.9 AB	2.2 ± 0.2 A	3.2 ± 0.2 A	52.9 ± 0.5 A	142 ± 8.5 C	72 ± 4.2 B

* Below the quantification limit.

## Data Availability

The datasets generated for this study are available on request from the corresponding author.

## Supplementary Materials

The following supporting information can be downloaded at: https://www.mdpi.com/article/10.3390/microorganisms10030562/s1, Table S1: Significant changes (*p* < 0.05) of lactic acid bacteria (LAB) concentrations (log CFU/g), pH, and a_w_ values, and of *L. monocytogenes* (Lm) and *Salmonella* spp. (Ss) concentrations (log CFU/g) analyzed during the manufacturing process of Milano-type salami made with different curing solutions. Values are means ± standard deviation of two replicate samples from Batch A (basic recipe), Batch B (vegetable nitrates), and Batch C (synthetic nitrates/nitrites). For each parameter, means with different lowercase letters within a column are significantly different (*p* < 0.05) during the process, while means with different uppercase letters within a row are significantly different (*p* < 0.05) among batches; Figure S1: Temperature profiles monitored for Batch A (basic recipe), Batch B (vegetable nitrates) and Batch C (synthetic nitrates/nitrites) during the process of Milano-type Salami
