# Supplementary material for: Inactivation of Listeria monocytogenes and Salmonella spp. in Milano-Type Salami Made with Alternative Formulations to the Use of Synthetic Nitrates/Nitrites
Source: Microorganisms. 2022 Mar 4;10(3):562. doi: 10.3390/microorganisms10030562 (PMC8953279; doi:10.3390/microorganisms10030562)
Supplement: Supplementary file 1 [file microorganisms-10-00562-s001.zip › microorganisms-1610252-supplementary.pdf]

Table S1. Significant changes ($p < 0.05$) of lactic acid bacteria (LAB) concentrations (log CFU/g), pH, and a_w values, and of *L. monocytogenes* (Lm) and *Salmonella* spp. (Ss) concentrations (log CFU/g) analyzed during the manufacturing process of Milano-type salami made with different curing solutions. Values are means ± standard deviation of two replicate samples from Batch A (basic recipe), Batch B (vegetable nitrates), and Batch C (synthetic nitrates/nitrites). For each parameter, means with different lowercase letters within a column are significantly different ($p < 0.05$) during the process, while means with different uppercase letters within a row are significantly different ($p < 0.05$) among batches.

Parameters	Time (days)	Batch A	Batch B	Batch C
Lab	0	8.1 ± 0.1aA	8.0 ± 0.1aA	8.1 ± 0.2aA
	1	8.0 ± 0.1aA	8.1 ± 0.2aA	8.1 ± 0.1aA
	3	8.6 ± 0.1bA	8.3 ± 0.1abA	8.7 ± 0.3aA
	4	8.6 ± 0.1bA	8.3 ± 0.1abA	8.6 ± 0.2aA
	7	8.4 ± 0.1bA	8.5 ± 0.2abA	8.5 ± 0.1aA
	15	8.8 ± 0.1cA	8.8 ± 0.1bA	8.3 ± 0.1aB
	30	8.7 ± 0.1bcA	8.4 ± 0.2abAB	8.1 ± 0.1aB
	45	8.5 ± 0.2bAB	8.3 ± 0.1abA	8.3 ± 0.1aA
	60	8.4 ± 0.1bA	8.5 ± 0.1abA	8.3 ± 0.2aA
	70	8.4 ± 0.1bA	8.4 ± 0.3abA	8.3 ± 0.1aA
pH	0	5.8 ± 0.1aA	5.7 ± 0.1aA	5.7 ± 0.1aA
	1	5.6 ± 0.1bA	5.7 ± 0.1aA	5.0 ± 0.1bB
	3	5.0 ± 0.1cA	5.0 ± 0.1bcA	4.8 ± 0.1cB
	4	5.1 ± 0.1cA	4.8 ± 0.1bB	4.8 ± 0.1cB
	7	5.1 ± 0.1cA	4.8 ± 0.1bBC	4.8 ± 0.1cC
	15	5.2 ± 0.1cA	4.9 ± 0.1bB	5.0 ± 0.1bB
	30	5.2 ± 0.1cA	4.9 ± 0.1bB	5.1 ± 0.1bA
	45	5.3 ± 0.1cA	5.0 ± 0.1cB	5.2 ± 0.1bC
	60	5.5 ± 0.1abA	5.2 ± 0.3bA	5.2 ± 0.1bA
	70	5.6 ± 0.1abA	5.0 ± 0.1bB	5.2 ± 0.1bB
a_w	0	0.97 ± 0.01aA	0.97 ± 0.01aA	0.97 ± 0.01aA
	1	0.96 ± 0.01aA	0.97 ± 0.01abAB	0.97 ± 0.01abA
	3	0.96 ± 0.01aA	0.97 ± 0.01abB	0.96 ± 0.01abB
	4	0.96 ± 0.01aA	0.96 ± 0.01abB	0.96 ± 0.01abB
	7	0.96 ± 0.01abA	0.95 ± 0.01bcA	0.95 ± 0.01bA
	15	0.94 ± 0.01bcA	0.95 ± 0.01cA	0.95 ± 0.01bA
	30	0.93 ± 0.01cdA	0.94 ± 0.01cdAB	0.94 ± 0.01bB
	45	0.92 ± 0.01deA	0.92 ± 0.01deA	0.92 ± 0.01cA
	60	0.91 ± 0.01eA	0.91 ± 0.02eA	0.92 ± 0.01cA
	70	0.91 ± 0.01eA	0.90 ± 0.01eA	0.91 ± 0.01dA
Lm	0	5.2 ± 0.3aA	5.5 ± 0.3aA	5.2 ± 0.1aA
	1	5.1 ± 0.2aA	5.5 ± 0.1aA	5.1 ± 0.1aA
	3	4.2 ± 0.1bA	4.5 ± 0.1bA	4.2 ± 0.1bA
	4	4.3 ± 0.1bA	4.4 ± 0.1bcA	3.8 ± 0.1bcB
	7	4.3 ± 0.2bA	4.4 ± 0.1bcA	3.9 ± 0.1bcB
	15	4.3 ± 0.2bA	4.2 ± 0.2bcA	3.5 ± 0.1cB
	30	4.0 ± 0.1bcA	4.1 ± 0.1bcA	3.5 ± 0.1cdB
	45	3.9 ± 0.2bcA	3.6 ± 0.5cdAB	3.1 ± 0.2dB
	60	3.8 ± 0.1bcA	3.7 ± 0.3cdA	3.2 ± 0.1dA
	70	3.6 ± 0.1cA	3.4 ± 0.1dB	2.9 ± 0.1eC
Sa	0	4.8 ± 0.3aA	5.3 ± 0.3aA	5.1 ± 0.1aA
	1	4.9 ± 0.2aAB	5.1 ± 0.2aAB	4.8 ± 0.1aB
	3	4.1 ± 0.1aA	4.2 ± 0.1bA	2.7 ± 0.1bB
	4	4.1 ± 0.2aA	3.6 ± 0.2cAB	3.1 ± 0.1cB
	7	3.9 ± 0.4abA	2.5 ± 0.2dB	3.1 ± 0.3cAB

15	3.9 ± 0.1abA	2.5 ± 0.3eB	3.0 ± 0.1cB
30	2.9 ± 0.1bA	0.7 ± 0.1fB	2.2 ± 0.1dC
45	3.1 ± 0.2bA	0.7 ± 0.1fB	1.8 ± 0.1eC
60	2.4 ± 0.6bcA	0.7 ± 0.1fB	2.4 ± 0.1fA
70	1.9 ± 0.1cA	0.7 ± 0.1fB	0.7 ± 0.1gB

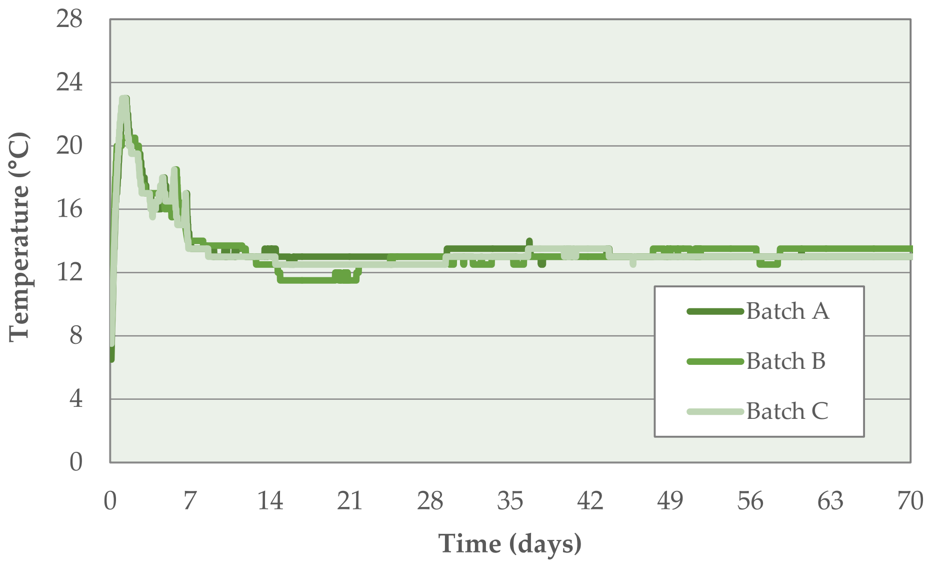

Figure S1. Temperature profiles monitored for Batch A (basic recipe), Batch B (vegetable nitrates) and Batch C (synthetic nitrates/nitrites) during the process of Milano-type Salami.
